# Predicted resistance to broadly neutralizing antibodies (bnAbs) and associated HIV-1 envelope characteristics among seroconverting adults in Botswana

**DOI:** 10.1038/s41598-023-44722-2

**Published:** 2023-10-24

**Authors:** Natasha O. Moraka, Wonderful T. Choga, Marea N. Pema, Moses Kudzai Chawawa, Irene Gobe, Margaret Mokomane, Ontlametse T. Bareng, Lynette Bhebhe, Nametso Kelentse, Graceful Mulenga, Molly Pretorius Holme, Terence Mohammed, Catherine K. Koofhethile, Joseph M. Makhema, Roger Shapiro, Shahin Lockman, Sikhulile Moyo, Simani Gaseitsiwe

**Affiliations:** 1 Botswana Harvard AIDS Institute Partnership, Bontleng, Private Bag BO320, Gaborone, Botswana; 2 School of Allied Health Professions, Faculty of Health Sciences, University of Botswana, Gaborone, Botswana; 3 Harvard T. H. Chan School of Public Health, Harvard University, Boston, MA USA

**Keywords:** Computational biology and bioinformatics, Infectious diseases

## Abstract

We used HIV-1C sequences to predict (in silico) resistance to 33 known broadly neutralizing antibodies (bnAbs) and evaluate the different HIV-1 Env characteristics that may affect virus neutralization. We analyzed proviral sequences from adults with documented HIV-1 seroconversion (N = 140) in Botswana (2013–2018). HIV-1 *env* sequences were used to predict bnAb resistance using bNAb-ReP, to determine the number of potential N-linked glycosylation sites (PNGS) and evaluate Env variable region characteristics (VC). We also assessed the presence of signature mutations that may affect bnAb sensitivity in vitro. We observe varied results for predicted bnAb resistance among our cohort. 3BNC117 showed high predicted resistance (72%) compared to intermediate levels of resistance to VRC01 (57%). We predict low resistance to PGDM100 and 10-1074 and no resistance to 4E10. No difference was observed in the frequency of PNGS by bNAb susceptibility patterns except for higher number of PNGs in V3 bnAb resistant strains. Associations of VC were observed for V1, V4 and V5 loop length and net charge. We also observed few mutations that have been reported to confer bnAb resistance in vitro. Our results support use of sequence data and machine learning tools to predict the best bnAbs to use within populations.

## Introduction

Evolution in antigenic diversity across HIV-1 subtypes is one of the main challenges in the development of antibody-mediated strategies for HIV prevention, treatment, and cure^1,2^. Recent attempts to find an HIV cure include the evaluation of broadly neutralizing antibodies (bnAbs) that may help eliminate HIV-1 infection^3^. Similarly, the use of broadly neutralizing antibody for prevention of HIV-1 infection or inhibition of transmitted founder viruses remains one of the main goals for using bnAbs as immunotherapy^4^. Some studies have shown effective bNab activity in animal models and some such as 2F5, 4E10, PGT121, VRC01, V3/N332, N123-VRC34.01, 3BNC117 and 10-1074 are still being tested in several clinical trials^5–8^.

One of the major hindrances to the effectiveness of these antibodies in neutralizing HIV-1 strains (whether in vitro or in vivo) is the presence of escape mutations in the HIV-1 envelope (Env) protein, which is the target for all bnAbs^3,5,6^. HIV *env* region is the most diverse compared to other viral proteins, it is important to assess for signature mutations that mediate escape in the transmitted founder virus and may limit the clinical use of bnAbs in high HIV-1 burdened populations. Similarly, the variability/diversity of HIV-1 *env* gene may pose a barrier to bnAb binding efficiency^9^. Several studies have evaluated HIV-1 Env characteristics to determine barriers to the ability of various bnAbs to neutralize different HIV subtypes^9,10^. Few studies have looked into how this diversity has evolved over time to show vast differences from within host (over 20% amino acid differences) to population scale differences, especially in settings of highly prevalent viruses such as HIV-1 subtype C (HIV 1 C)^11,12^.

For resource limited settings such as Botswana, it is difficult to carry out in vitro assays to detect neutralization capacity, and direct clinical trial data in the country to study bnAb efficacy is limited. However, machine learning prediction models/tools in silico can determine potential resistance or susceptibility of bnAbs in this setting^13,14^. Correlation of genotypic viral sequence data with actual in vitro data helps us determine the strength of these prediction models^15^. Because HIV-1 strains vary in their sensitivity to antibodies, accurate prediction of the sensitivity can be used to find the best bnAb combinations that can potentially be used to neutralize regional HIV strains^13^. In this analysis, we used proviral HIV-1C sequences from adults with documented HIV-1 seroconversion to predict (in silico) resistance to 33 known bnAbs and evaluate the different HIV-1 *env* sequence characteristics that may affect virus neutralization in Botswana.

## Results

A total of 140/6078 (2.3%) of consensus proviral sequences from adults with documented seroconversion from the BCPP project were included in this analysis. One-hundred-and six (76%) seroconverters were ART-naïve at the time of sample collection with a median viral load of 3.5 (Q1, Q3: 1.6, 4.3) log10 copies/mL. Median age was 26 years (Q1, Q3: 22, 32) and most participants (81%) were female (Table 1).

**Table 1:** Baseline demographics of adults with documented HIV-1 seroconversion in the Botswana combination prevention project (BCPP).

Baseline characteristics (N = 140)	
Age (years), median (IQR)	26 (22, 32)
Gender, n (%)	
Female	113 (80.7)
Male	27 (19.3)
HIV RNA load (log10 copies/mL), Median (IQR)	3.5 (1.6, 4.3)
Marital status, n (%)	
Married	10 (7.2)
Single	130 (92.8)
ART status, n (%)	
ART naive	106 (75.7)
ART experienced	34 (24.3)
Highest education level, n (%)	
Primary/non formal	23 (16.4)
Junior secondary	67 (47.9)
Senior secondary	37 (26.4)
Tertiary	13 (9.3)
Intervention, n (%)	
Standard of care	54 (38.9)
Study intervention arm	86 (61.4)
Employed, n (%)	
No	105 (75.0)
Yes	35 (25.0)

This table describes the baseline characteristics of the adults with documented seroconversion from the BCPP study.

*ART* antiretroviral treatment, *CD4* cluster of differentiation 4 T-cells, *IQR* interquartile range.

### Predicted bnAb resistance among adults with seroconversion

**Figure 1:**
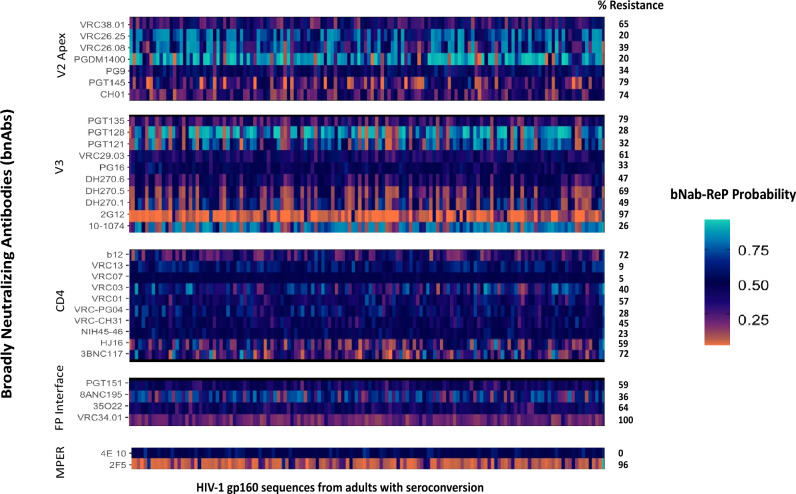
Predicted bNAb resistance based on bNab-ReP predictions from HIV-1 gp160 proviral sequences from seroconverters in Botswana. bNab-ReP algorithm was used to predict resistance/sensitivity of 140 HIV-1 gp120 sequences aligned to HXB2 and consensus HIV-1C reference from proviral strains of adults with documented seroconversion in Botswana. Probability gradient indicates probable sensitivity/resistance where values lower than 0.5 indicate probable resistance (dark orange color being most resistant) and vice versa. Percentage (%) resistance indicates percentages of resistance using cutoff bNab-ReP probabilities of 0.5.

The prevalence of predicted resistance to 1 or more bNAbs was variable among seroconverters (Fig. 1). High rates (between 70 and 100%) of resistance were observed for other bnAbs including VRC34.01, CH01, PGT145, PGT135, 2G12, b12, 3BNC117 and 2F5. Intermediate rates of resistance were observed for VRC01 (57%) and low resistance was predicted for 10-1074 (26%). Low rates of resistance (< 20%) were also predicted for PGDM1400, VRC26.25. Similarly, very low rates of resistance (< 10%) were predicted for VRC07 and VRC13. We observe mixed frequencies of resistance across bnAb classes, for example, a sharp contrast is observed in MPER binding bnAbs 2F5 (96%) and 4E10 (0%). Similarly, although V3 binding bnAbs have resistance frequencies going up to 97% for 2G12 but PGT121, PGT128, PG16, and 10-1074 show rates less than 40%.

### Frequency and quantity of gp120 potential N linked glycosylation sites (PNGS)

**Figure 2:**
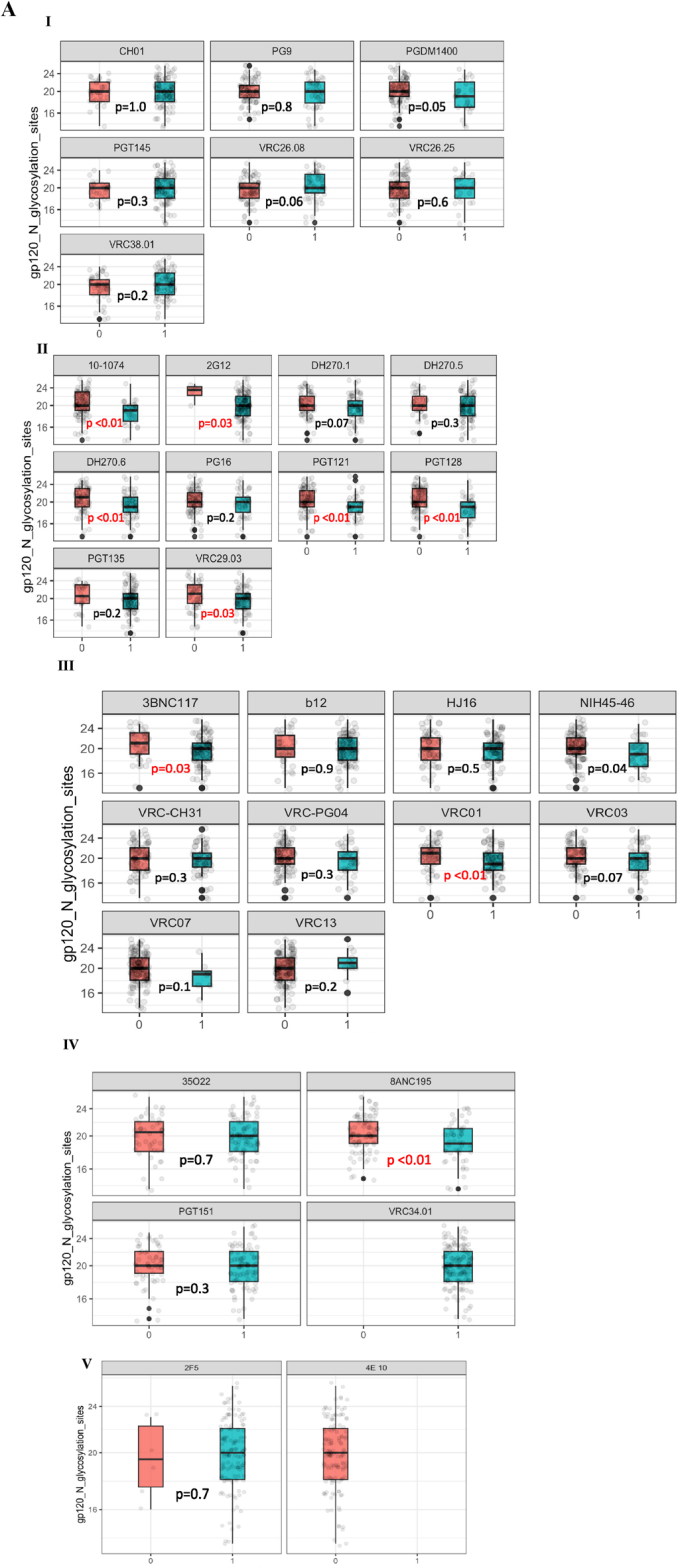

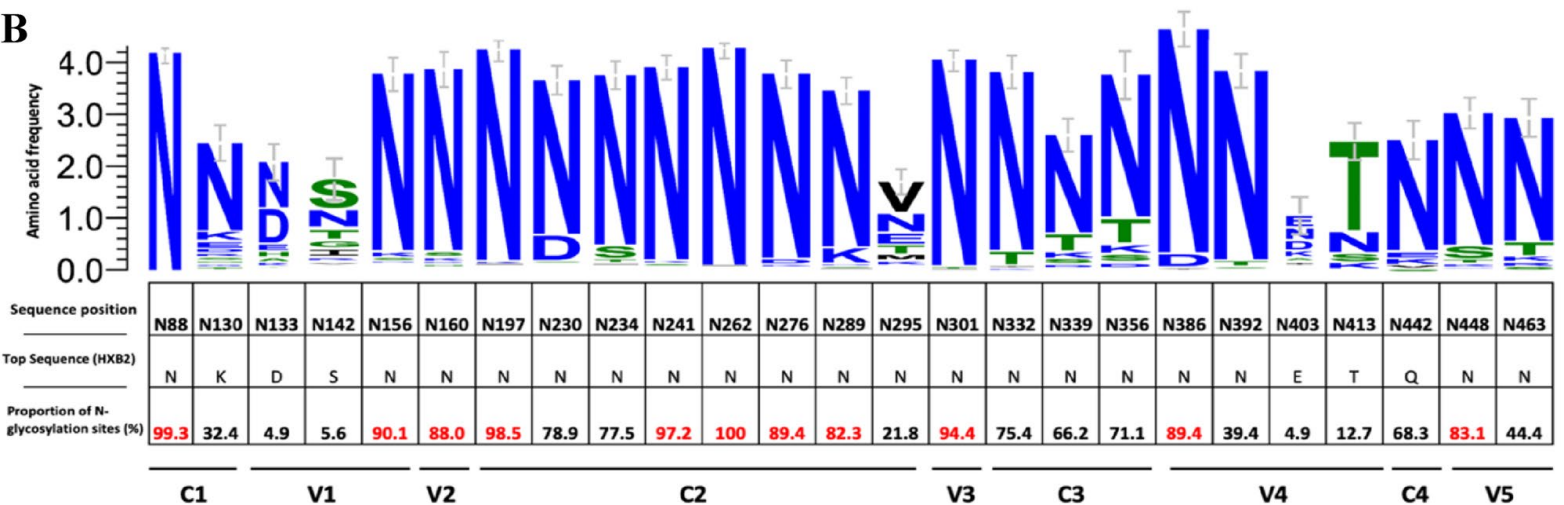
Frequency of gp120 *N*-glycosylation sites frequencies. This figure shows differences in overall number of PNGS across all sequences. (**A**) I–V represent V2 apex binding antibodies, V3 binding antibodies, CD4 binding antibodies, FP interface binding antibodies as well as MPER binding antibodies, respectively. These proportions were plotted for strains with predicted resistance (1/blue) and those with predicted susceptibility (0/pink). P values indicate differences in proportions calculated using the Wilcoxon ranksum test. (**B**)Percentage abundance of signature PNLG sites (N88–N463). The logo section of the graph shows the proportions of dominant amino acids from all consensus proviral sequences at each PNG site, the size of the letter represents the proportion of sequences with that amino acid among all sequences. These sites are divided by their specific PNG domains.

A median of 20 (Q1, Q3: 18, 22) PNGS were observed among all sequences evaluated. No difference was observed in frequency of PNGS compared by bnAb resistance/sensitivity in V2 apex and MPER binding bnAbs (Fig. 2). For V3 binding bnAbs; 10-1074, 2G12, DH270.6, PGT121, PGT128, VRC29.03 resistant strains showed significantly higher number of overall PNGS compared to sensitive strains. We further evaluated the abundance of 25 well characterized PNGS (adapted from Sutar et al.^9^) among all HIV *env* sequences divided by their corresponding functional domains (Fig. 2). These proportions were plotted along with sequence variations among each signature site. High abundance of above 80% of PNGS was observed in several highly conserved signature PNG sites: N88, N156, N160, N197, N241, N262, N276, N289, N301, N386, N448 (Fig. 2).

### HIV Env gp120 variable loop length and net charge (V1–V5)

We analyzed variable loop lengths including V1–V5 (including hypervariable regions) length and net charge for all gp120 sequences. The differences observed between the envelope characteristics were calculated using Wilcoxon Ranksum Test, stratified by predicted resistance/sensitivity.

Although these regions are characterized with extreme variability for HIV-1C variants, not many differences were observed when looking at V1–V4 loop lengths, except for VRC26.25 where V1 loop (and V1 hypervariable loop region, data not shown) had shorter loop lengths observed in resistant strains (*p* < 0.01). A similar observation was observed for NIH45–46 resistant strains showing lower V4 loop region (*p* = 0.04). The most significant differences in variable region correlations were observed with V5 loop length, where CD4 bnAb 2G12, DH270.5 and FP interface 35O22 resistant strains had shorter V5 loop lengths; *p* = < 0.01, < 0.01 and 0.02 consecutively. Boxplot representing comparison of medians with *p* values are provided in Fig. S1.

Net charge was generally observed to be similar when comparing predicted resistant/sensitive strains. V1 charge distributions were observed to be significantly different among in VRC26.25 (*p* = 0.01), PGT121 (*p* < 0.01) and VRC01 (*p* = 0.01). A strong association was also observed for 2F5 resistant strains (*p* < 0.01); however, this was the only significant observation made for this bnAb for all sequence characteristics (Fig. S1). V4 charge distributions were observed to vary only among CD4 binding bnAbs HJ16 and VRC03 (*p* = 0.02 for both).

### Signature amino acid positions and mutations

The presence of signature amino acid mutations on key positions was evaluated across all alignments with high level predicted resistance (70–100%), excluding FP interface bnAbs. We identified signature mutations which confer resistance to a subset of HIV-1C bnAbs from Bricault. et al.^16^; who identified signature patterns by correlating each amino acid and PNGs across several HIV *env* alignments from different HIV subtypes based on available neutralization data to V2 apex, V3, CD4 and MPER binding bnAbs. Frequencies of mutations observed in HIV-1 subtype C alignments to several bnAbs were evaluated as percentages and correlated with either predicted resistance or sensitivity by comparison of proportions test, where *p* values of 0.05 were considered statistically significant. All positions of interest were associated with bnAb resistance.

#### V2 apex mutations

Signature amino acid positions associated with proven in vitro resistance to CH01 and PGT145 were evaluated by correlating frequency of mutation to predicted resistance (Table S1a). We observed high associations across key signature sites to CH01, PGT145. The common loss of K169 and K171 which are associated with some V2 apex binding bnAbs were not observed across any of the resistant strains. E164 was observed and associated with resistance to both CH01 and PGT145. N160 was associated with resistance to most V2 apex binding bnAbs but was observed in sequences predicted to be sensitive to PGDM1400 (data not shown). We did not observe the K169E/T mutation in high frequencies among sequences with high predicted resistance.

#### V3 mutations

Most V3 signature sites are generally well conserved (Table S1b). Positions 332, 334 which are usually associated with resistance to most V3 binding were associated with resistant strains; 100% of resistant strains for having an asparagine (N) at position 334 for both 2G12 and PGT135. A few sites had several variations, but these were all significantly associated with resistance: 295, 336, 413, 640, 774.

#### CD4 mutations

Majority of CD4 resistant mutations are often observed around the CD4 binding site. We observe the mutation T234N which is a non-contact mutation, this appeared as a consensus amino acid among our sequences and was present in 126/140(90%) of our sequences; were 93 sequences were resistant to 3BNC117 and 91 to b12. We did not observe the mutation G458Y and D99Y was observed in very low (< 5%) frequencies. Position 371 had amino acids I (consensus) and V (mutation) observed in majority of sequences, this is different from the T371K mutation usually observed for subtype C viruses. We also observe a few other mutations that were associated with high levels of resistance across most bnAbs: S364H/P and G471A.

#### MPER mutations

We evaluated signature amino acid positions associated with 2F5 resistance. Overall, all positions considered had strong associations with resistance including positions where only wildtype amino acids were represented higher than mutations. We did not record mutations that are associated with complete MPER binding bnAb resistance; N671T, W672L, WG80G and F673L among all 2F5 resistant strains. We did however have K683R mutation observed in 25/140 (18%) of the sequences, 24 of which were to resistant strains to 2F5 (Table S1d). Of note this mutation was also observed among all the 4E10 sensitive strains (data not shown).

## Discussion

We report, for the first time, prediction of resistance to 33 different bnAbs using HIV-1 proviral sequences from adults with HIV-1 seroconversion in Botswana, using a readily available in silico approach (bNab-ReP)^15^. There are limited data on the diversity of HIV-1 *env* and how it relates to probability of the successful use of bnAbs as immunotherapies in sub-Saharan African countries. We here report on the variability of the HIV Env characteristics that may affect susceptibility and potential use of these bnAbs in Botswana, including signature mutations that may facilitate bnAb escape in the setting of HIV-1 subtype C.

Generally, predicted bnAb resistance varied among different bnAbs even if they were of the same binding type class, indicating variability in the potential neutralization capabilities of different bnAbs in the HIV-1C setting. For instance, we observe high rates of predicted resistance to 2F5 and no resistance to 4E10 among the two evaluated MPER binding site bnAbs. Similarly, the prediction of resistance also varied among other classes, including V2 apex and V3 binding bnAbs. Consistent with other studies that have analyzed phenotypic neutralization of HIV-1C envelopes^9,17^, we observed diversity in the predicted response to bnAbs that target V2. Recently, Mandizvo et al.^17^ demonstrated differences in neutralization breath of different bnAbs; 2G12 (23%) compared to 10-1074 (80%) among V3 binding bnAbs, as well as 3BNC117 (59%) compared to VRC07-LS (100%) among CD4 binding bnAbs in single *env* genomes from nine South African individuals followed longitudinally post-acute HIV-1 infection. The authors also describe that transmitted founder viruses were observed to be more resistant to VRC01 and sensitive to PGDM1400, but a reverse observation is seen for more chronic infections, and we observed similar results for adults with documented seroconversion. In Botswana, several studies have evaluated VRC01 and 10-1074 bnAbs in both adult and infant populations showing moderate to good neutralization breath, especially for 10-1074^18,19^; we demonstrate that the prediction model of choice was able to predict similar results in the setting of adults with HIV-1 seroconversion, further highlighting the potential use of machine learning and artificial intelligence to determine bnAbs to use in different clinical settings. Furthermore, the results observed in our study show moderate to low levels of resistance in bnAbs used in germline targeting vaccines like VRC01, VRC26.25, PG9, PGT 121 and PGT128. These results could support the use of these bnAbs as templates for potential vaccine development^20,21^ within the sub-Saharan African region, with caution against ongoing epitope changes and bnAb recognition and binding site altering mutations due to high viral diversity of the predominant HIV-1 subtypes.

Assessing N-linked glycans found in PNG sites in HIV-1 is important as they take up an average 50% of total mass of gp120 and are used by the virus to escape bnAb neutralization and increase viral pathogenesis^22^. Our data reveal high conservation of signature PNG sites across HIV-1C sequences, and higher levels of PNG sites were mostly associated with V3 binding bnAbs. We observed highly conserved PNG sites: N88, N156, N160, N197, N241, N262, N276, N289, N301, N386, N448. These data agree with results from Sutar et al.^9^, who evaluated the geospatial differences in HIV-1C genetic signatures and what attributes differentiate region-specific HIV-1C with virus neutralization to key bnAbs using full-length sequences across 37 different countries (including Botswana) retrieved form the Los Alamos National Laboratory HIV database. They reported that N88, N156, N160, N197, N276, N301 and N386 were observed to be highly conserved in all countries with over 70% abundance, where N301 was the most conserved (89–100%). Similarly, in our cohort, N301 conservation was 95% among our sequences, with N262 as the most conserved (100%) PNG site which has been previously predicted to interact with PGT151^23^. Furthermore, Sutar et al.^9^ also observed significant differences in PNG sites from India and South Africa, where N130, N295, N392 and N448 which represent C1,C2,V4 and C4, these sites are responsible for the integrity of the mannose patch as well as associations with bnAbs such as 2G12, VRC-PG05 and PGT135^22^. We observe low conservation at PNG site N295, which is one of the sites where glycans that interact with V3 bnAbs are often positive for neutralization; this supports our finding of significant differences between resistant and sensitive strains within the V3 binding bnAbs. However, the extreme variability of V3 positions 336–442 may lead to inconclusive observations in terms of bnAb sensitivity^16^. We also observe fewer PNGS in most V3 resistant strains compared to sensitive strains. This is in contrast with what has been described in other studies but could be attributed to the effects of evolutionary pressure that lead to high diversity of HIV-1C. Naturally, glycan holes can create vulnerable sites on the virus that are more susceptible to antibody binding and neutralization, inducing autologous neutralizing responses^24,25^. The V3 loop glycan shield is the most conserved of the variable regions and this evolutionary pressure can therefore cause mutations in the glycosylation sites as a defense against glycan shielding, which in turn would lead to poorer antibody recognition.

Traditionally, variable region characteristics of HIV Env have been used to inform HIV vaccine designs, where the higher variation requires a robust and potent intervention. Our results report minor differences in variable characteristics by predicted bnAb resistance, although these differences which were most likely to affect bnAb sensitivity were observed in V1, V4 and V5. The variations observed in these sites have been reported to have impact on neutralization breath of majority of different bnAbs, this has been the main driver for development of bnAb cocktails targeting different regions of the *env* gene for treatment and prevention of HIV-1^26–28^.

The presence of mutations in majority of sequences with high levels of predicted bnAb resistance has been a general observation for several other studies^5,16,17^. We observe similar results to what has been observed in different studies done both in *vitro* and in silico^29^, where introduction of several mutations in the *env* backbone of most HIV-1 pseudoviruses has led to reduced potency and neutralization breath of several bnAbs. We also reported the T234N which has been a glycosylation site associated with resistance to several CD4 binding bnAbs^16,30,31^, including strong associations with HIV-1 vertical transmission^32^.

Our study had several limitations. We used the bNab-ReP tool to predict HIV-1 resistance/sensitivity to 33 different known bnAbs. Although the tool has been shown to have a great correlation between in silico predictions and in vitro data through the CATNAP database (96% prediction accuracy)^15^, several newer algorithms have been deposited in Github which may yield more accurate results by updating the tools with other predictors selected apriori through time^13^. Although this is a limitation, it also highlights the need to develop within-country, regional and subtype-specific algorithms for such predictions with the hope of getting predictions that are able to be used across all subtypes and easier to perform by end-users in resource-limited countries. We only evaluated a subset of mutations for a subset of bnAbs of interest, and future studies will include HIV-1C-specific mutations that correlate with phenotypic data demonstrating actual resistance caused by these mutations. Another limitation is the lack of phenotype data from Botswana or from our current cohort of interest, as we are unable to correlate genotypic prediction data with in vitro neutralization which may allow us to draw more firm conclusions. The strengths of our analyses include the ability to use a readily available prediction tool using sequences from recent seroconversions in Botswana. The predictions of resistance and sensitivity/susceptibility shown in our results are in line with what has already been published for teams that used phenotypic assays within the region to detect resistance to several bnAbs.

## Conclusions

We report moderate to high levels of predicted bnAb resistance among HIV-1C proviral sequences of people with documented recent seroconversion in Botswana, and high variability of HIV-1C Env. Our results further highlight the need to analyze sequence data and use prediction tools to determine the best bnAb combinations to use in the setting of highly variable HIV-1C infection.

## Methods

### Study design and population

We analyzed HIV-1 consensus proviral sequences from adults with documented HIV-1 seroconversion (N = 140) who were enrolled in a previous population-based household study (Botswana Combination Prevention Project (BCPP), 2013–2018)^33^. In brief, the BCPP, which enrolled a total of 6078 participants, was a PEPFAR-funded, pair-matched, cluster-randomized trial that was designed to test if combination prevention interventions can reduce population-level cumulative 3-year HIV incidence. The study was conducted in 30 different paired communities matched using factors such as population size, pre-existing health services, the age structure of the population as well as geographic location and classification (urban vs rural). Seroconversion was documented within the duration of the study (2013–2018), where seroconversion was defined as the first positive HIV test after at least one negative baseline HIV test result.

### Sequence generation and subtyping

HIV-1 near-full length proviral sequences (nFLs) included in this analysis were previously generated using proviral DNA as a template for a modified long-range HIV genotyping protocol which has been described in full elsewhere^34,35^. All nFLs were analyzed to determine the subtypes using online tools REGA HIV-1 subtyping tool, version 3^36^ and COMET^37^ and adjusted for hypermutations using Hypermut (http://www.hiv.lanl.gov/)^38^. We selected HIV-1 (nFLS) from participants with documented HIV-1 seroconversion (n = 140) within the duration of the study, nFLS were aligned with HXB2 (GenBank: K03455.1) and a consensus HIV-1C reference generated from all BCPP nFLS, nFLS using MUSCLE software. The quality of the sequences was assessed prior to trimming the multiple sequence alignment to gp160 (HXB2 position 127–856) and gp120 (127–475) regions using AliView 1.26. gp160 and g120 were used for all the downstream analyses.

### Prediction of bnAb resistance

All HIV gp160 region sequences (n = 140) from participants with documented seroconversion plus HXB2 and HIV-1C consensus were used to predict the sensitivity of contact sites to 33 known bNAbs using the bNAb-ReP algorithm (https://github.com/RedaRawi/bNAb-ReP)^15^. These bnAbs include those targeting all 6 known binding sites: the V2 trimer apex, V3 supersite, CD4 binding site, Fusion peptide and gp120/gp41 interface, as well as the membrane-proximal external region (MPER) sites. A cutoff of 0.5 probability values generated from the bNab-ReP tool was used to classify sensitivity (≥ 0.5) or resistance (< 0.5).

### HIV Env characteristics and signature mutations analysis

All gp120 alignments were used to determine the number of potential N-linked glycosylation sites (PNGS) and compared between bNAb-resistant and sensitive strains using the *N*-glycosite tool in the Los Alamos National Laboratory (LANL) HIV sequence database (hiv.lanl.gov). Alignments were also used to determine the full variable loop lengths [V1 (130–158 by HXB2 numbering), V2 (157–197), V3 (295–332), V4 (385–418) and V5 (459–470)] and net charge patterns using the ‘Variable Region Characteristics” tool in the LANL HIV sequence database (hiv.lanl.gov). Mutations conferring resistance to known bnAbs were assessed a priori, using published data^16^.

### Statistical analysis and data representation

Descriptive statistics were presented as percentages with 95% confidence intervals or medians with interquartile ranges. Predictors of specific outcomes were examined using paired t-tests and Wilcoxon ranksum tests for assessment of variables between predicted bnAb resistance or sensitivity, we did not further perform multivariate analysis for adjusted comparisons. Stata version 15 and R packages were used for statistical analysis and graph generation. *p* values less than < 0.05 were considered as significant.

Heatmap was produced using “Heatmap” package in R.

Boxplots describing variable region characteristics stratified by predicted bNAb resistance were generated using “ggplot” and “facet_wrap” packages using R v5.5.4 (2022.07.1)^39^.

Logo plots were used to determine proportion of amino acids across each *n*-glycosylation site of interest using the weblogo tool^40^.

### Ethical approval statement

The BCPP study protocol, informed consent, and other materials were approved by the Botswana Health Research Development Committee (HRDC) and US Centers for Disease Control and Prevention Institutional Review Boards. BCPP participants gave written informed consent and for participants aged 16–17 years, written informed assent was provided with permissions from parents or guardians. The study is registered at ClinicalTrials.gov (NCT01965470, study start date: 30/10/2013) and we confirm that all methods were performed in accordance with the Helsinki declaration following International Conference on Harmonization Good Clinical Practice (ICH GCP) guidelines.

### Supplementary Information

Supplementary Figure S1.

Supplementary Table S1.

## Data Availability

All data on performing bNAb_ReP can be found at https://github.com/RedaRawi/bNAb-ReP. Data describing BCPP participants can be found in previous publication at https://www.ncbi.nlm.nih.gov/pmc/articles/PMC6800102/. All other data supporting the findings of this study can be made available by the corresponding author SG.
