# Supplementary material for: Predicted resistance to broadly neutralizing antibodies (bnAbs) and associated HIV-1 envelope characteristics among seroconverting adults in Botswana
Source: Sci Rep. 2023 Oct 24;13:18134. doi: 10.1038/s41598-023-44722-2 (PMC10598268; doi:10.1038/s41598-023-44722-2)
Supplement: Supplementary file 1 — Supplementary Figure S1. [file 41598_2023_44722_MOESM1_ESM.docx]

**Figure S1: gp120 variable regions (V1-V5) loop lengths and net charge by predicted bnAb resistance**

Boxplots showing HIV-1C env characteristics; V1 to V5 loop lengths and net charge (A-E) in the y axis against predicted bnAb resistance (1/pink) or susceptibility (0/blue) for all 33 bnAbs of interest. bnAbs are grouped by binding site. P values indicated were generated by Wilcoxon Ranksum Test to compare characteristics by predicted resistance, p values less that 0.05 were considered significant and highlighted in red.
